# Comparison of Modic Changes in the Lumbar and Cervical Spine, in 3167 Patients with and without Spinal Pain

**DOI:** 10.1371/journal.pone.0114993

**Published:** 2014-12-15

**Authors:** Li Sheng-yun, Suyou Letu, Chen Jian, Maiwulanjiang Mamuti, Liu Jun-hui, Shan Zhi, Wang Chong-yan, Fan Shunwu, Fengdong Zhao

**Affiliations:** Department of Orthopaedics, Sir Run Run Shaw Hospital, School of Medicine, Zhejiang University, Hangzhou, 310016, P.R. China; University of Michigan, United States of America

## Abstract

**Background Context:**

There are few comparisons of Modic changes (MCs) in the lumbar and cervical spine.

**Purpose:**

Compare the prevalence of MCs in the lumbar and cervical spine, and determine how MC prevalence depends on spinal pain, age, disc degeneration, spinal level, and the presence or absence of kyphosis.

**Study Design:**

Retrospective clinical survey.

**Materials and Methods:**

Magnetic resonance images (MRIs) were compared from five patient groups: 1. 1223 patients with low-back pain/radiculopathy only; 2. 1023 patients with neck pain/radiculopathy only; 3. 497 patients with concurrent low-back and neck symptoms; 4. 304 asymptomatic subjects with lumbar MRIs; and 5. 120 asymptomatic subjects with cervical MRIs.

**Results:**

The prevalence of MCs was higher in those with spinal pain than in those without, both in the lumbar spine (21.0% vs 10.5%) and cervical spine (8.8% vs 3.3%). Type II MCs were most common and Type III were least common in all groups. The prevalence of lumbar MCs in people with back pain was little affected by the presence of concurrent neck pain, and the same was true for the prevalence of cervical MCs in people with neck pain with or without concurrent back pain. When symptomatic patients were reclassified into two groups (back pain, neck pain), the prevalence of lumbar MCs in people with back pain was greater than that of cervical MCs in people with neck pain. The prevalence of lumbar and cervical MCs increased with age, disc degeneration, (descending) spinal level, and increased kyphosis.

**Conclusions:**

There is a significantly higher prevalence of MCs in patients with back and neck pain. The reported association with increased kyphosis (flat back) is novel.

## Introduction

Degenerative changes of the vertebral endplate or “Modic changes” (MCs), have been considered important in low back pain since Modic first described them in magnetic resonance images (MRI) from patients with spinal degeneration [1]–[3]. Initially, two types were described and a third was defined subsequently. According to Modic, Type I changes appear hypointense on T1-weighted imaging (T1WI) and hyperintense on T2-weighted imaging (T2WI). Type I MCs are associated with fissuring of the cartilaginous endplate and increased vascularity or oedema within the subchondral bone marrow. Type II MCs appear hyperintense on T1WI and isointense or hyperintense on T2WI. Signal changes are less marked for Type II than for Type I, and reflect fatty degeneration of the bone marrow, or endplate necrosis. Type III MCs appear hypointense on both T1WI and T2WI and are assumed to represent subchondral bone sclerosis (Table 1). Previous studies of Modic changes have focused mainly on their pathogenesis and the clinical significance of changes between different types of MC, as well as their relationship with low back pain [4]–[8]. The main cause of MCs has been attributed to minor trauma of the endplate as a result of repetitive loading [9] or to recurrent disc injury which causes an inflammatory reaction within the nucleus pulposus, initiating endplate changes and subsequent back pain [10]. More recent findings suggest that levels of IL6, IL8 and PGE2 in the intervertebral disc are significantly higher when Modic Type I changes are present compared to Type II, suggesting that inflammatory changes in the disc may be involved in the initiation of Type I changes [11]. However, pathological changes in the spine are dynamic and the natural history of MCs suggests that they are reversible and this has raised doubts concerning the usefulness of these MRI changes as an indicator of clinical symptoms or surgical outcomes [12].

**Table 1:** Three types of Modic change.

Modictype	T1 signal	T2 signal	represents
**0**	Normal	Normal	Normal
**I**	LOW	HIGH	Vascularized bone marrow and/or edema
**II**	HIGH	Isointense orhyperintense	Proliferation of fatty tissue
**III**	LOW	LOW	Sclerotic bone

If Modic changes represent a progressive response to injury then it is possible that some types of Modic change may be more highly correlated with pain than others, especially if they are linked with inflammatory changes. Modic changes have been studied most extensively in the lumbar spine where they are most common [9]–[11], [13] and some studies have suggested strong links with pain and disc degeneration. [14] However, relatively little is known about the prevalence or distribution of MCs in other regions of the spine and whether they are associated with symptoms. The cervical spine, like the lumbar spine, is highly mobile and is prone to degeneration and pain. Furthermore, clinical evidence suggests that Modic changes are often observed in people with neck pain. The aim of this study, therefore, was to evaluate the distribution and prevalence of the different types of Modic change in the lumbar and cervical spine in people with back and/or neck pain and to determine how they are influenced by factors such as pain, age, degeneration, spinal level, and the presence of kyphosis.

## Materials and Methods

### Study Design

The medical records of patients attending the Sir Run Run Shaw Hospital over a 12 month period (Jan–Dec 2011) were reviewed to identify all patients who had back and/or neck pain. The MRI scans of these patients were then assessed to evaluate disc degeneration, sagittal spinal curvature and the presence and type of Modic changes in the lumbar and/or cervical spine. In order to investigate whether pain is associated with a higher prevalence of MCs, we also collected MRI scans from 424 asymptomatic patients who underwent physical examination in the authors’ hospital between January and December 2011. Patients with any pathological lesions, such as tumor, infection, fracture or congenital abnormality, were excluded. The study was approved by the Medical Ethics Committee of Sir Run Run Shaw hospital, and all patients gave written informed consent for their information to be stored in the hospital database and used for research.

### Patient populations

Patients were subdivided into five groups: 1. a “back pain” group comprising 1223 patients (650 males and 573 females) with a mean age of 45.3±12.7 yrs (range 22–85 yrs) who had low-back pain or sciatica only; 2. a “neck pain” group comprising 1023 patients (536 males and 497 females) with a mean age of 44.9±11.1 yrs (range 19–86 yrs) who had neck pain or radiculopathy only; 3. a “back and neck pain” group comprising 497 patients (281 males and 216 females) with a mean age of 44±9.3 yrs (range 22–73 yrs) who had both low-back pain and neck pain concurrently; 4. asymptomatic lumbar group comprising 304 patients (196 males and 108 females) with a mean age of 44.8±9.5 yrs (range 24–64 yrs) and 5. asymptomatic cervical group comprising 120 patients (40 males and 80 females) with a mean age of 43.3±10.2 yrs (range 24–64 yrs).

MRI scans of all patients in the three symptomatic groups were reviewed to determine the prevalence and types of MCs and investigate their associations with disc degeneration, disc level, age, and spinal kyphosis. Subsequently, symptomatic patients were reclassified into two groups. Group 6 comprised all patients with back pain, and Group 7 comprised all patients with neck pain. The prevalence of MCs in Groups 6 and 7 were investigated to determine whether the prevalence of MCs in the lumbar spine of low back pain patients is different from the prevalence of MCs in the cervical spine of neck pain patients. Groups 6 and 7 were also compared with the corresponding asymptomatic groups (4 and 5 respectively) to determine whether MCs are more prevalent in people with back and neck pain.

### MR image evaluation

All lumbar MR scans were performed on a GE Sigma CV/I (1.5 T) using a surface coil. Sagittal T1- and T2-weighted imaging sequences (T1WI and T2WI) were used to examine the lumbar spine. T1-weighted images were obtained using repetition/echo times of 420/13 msec and a bandwidth of 140 Hz/Px, while T2-weighted images used 2300/105 msec and 160 Hz/Px. The matrix size was 320×224 for both T1 and T2 images, with a field of view of 28×28 mm. Slice thickness and inter-slice gap were 4 mm and 1 mm respectively for both T1 and T2 images. For each imaged lumbar spine, nine sagittal slices were obtained for both T1WIs and T2WIs. For the cervical spine, T1- and T2-weighted sequences were obtained on the same machine using the same parameters as those used for the lumbar images, except that the slice thickness was set at 3.0 mm.

All MRIs were evaluated by two independent observers, an experienced radiologist and an orthopaedic surgeon, in order to determine the intra-observer and inter-observer reliability of these measurements.

#### Type of Modic change

**Figure 1:**
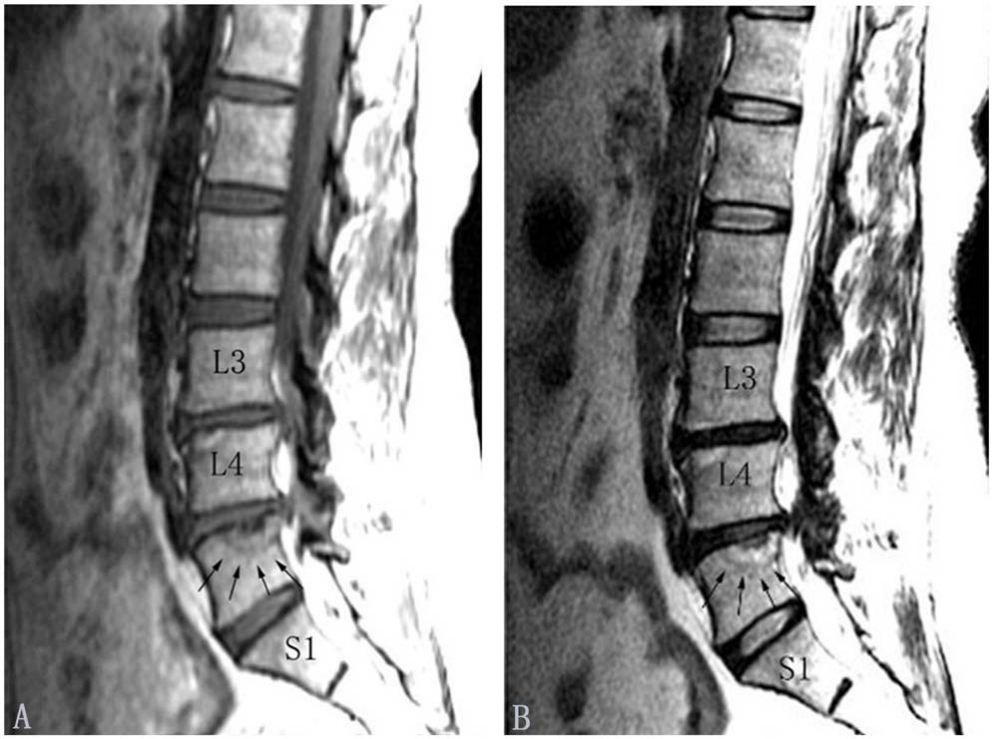
Modic type I changes in the lumbar spine (indicated by arrows). Modic type I changes appear hypointense on T1-weighted images (A) and hyperintense on T2-weighted images (B).

**Figure 2:**
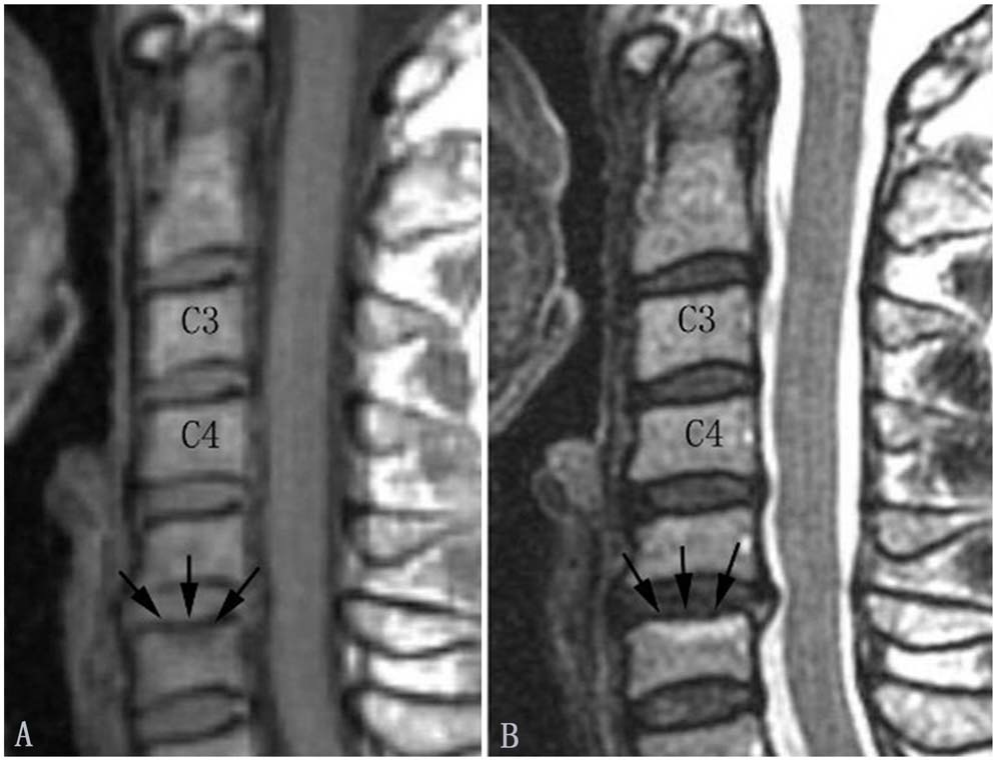
Modic type I changes in the cervical spine (indicated by arrows). Modic type I changes appear hypointense on T1-weighted images (A) and hyperintense on T2-weighted images (B).

**Figure 3:**
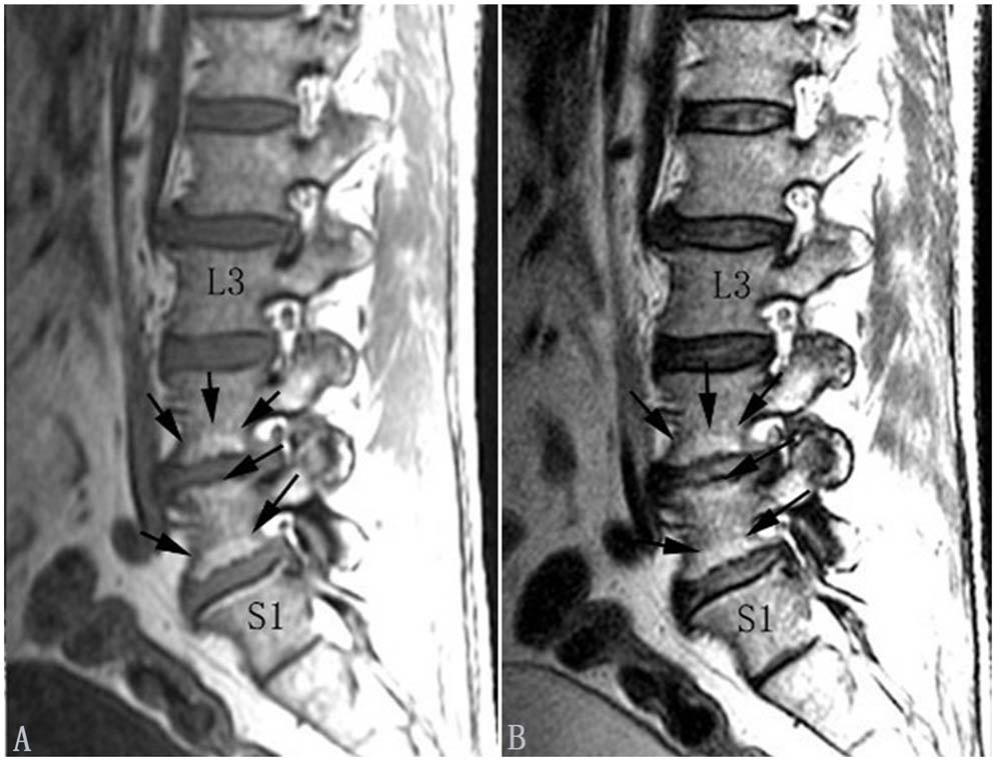
Modic type II changes in the lumbar spine (indicated by arrows). Modic type II changes appear hyperintense on T1-weighted (A) and isointense or hyperintense on T2-weighted images (B).

**Figure 4:**
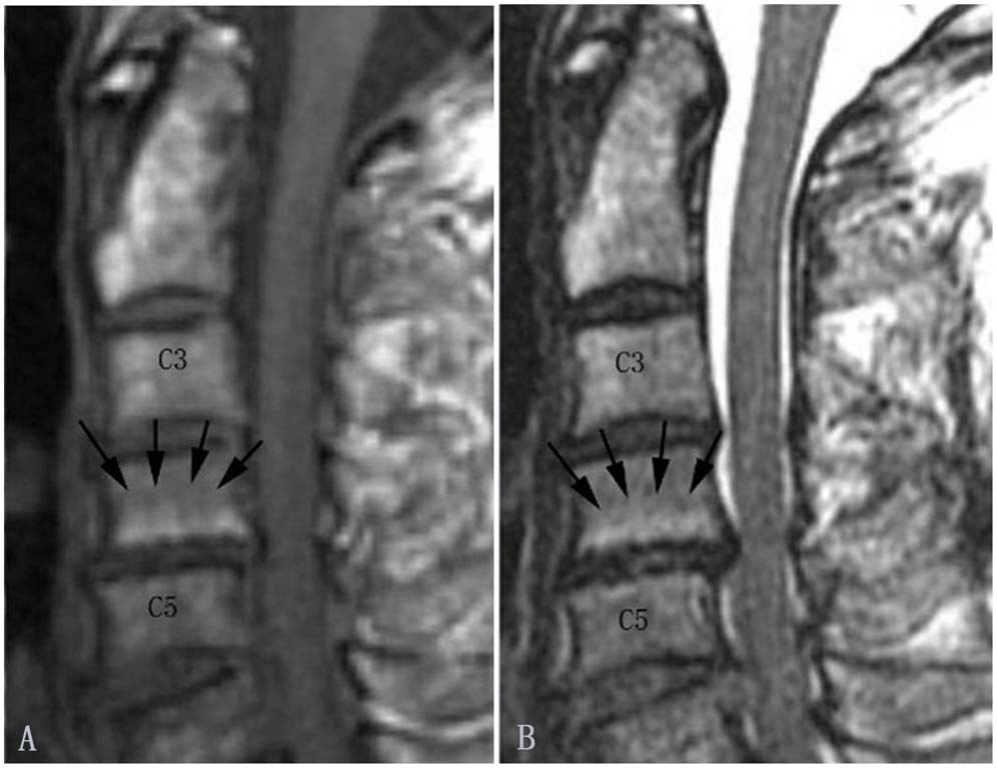
Modic type II changes in the cervical spine (indicated by arrows). Modic type II changes appear hyperintense on T1-weighted (A) and isointense or hyperintense on T2-weighted images (B).

**Figure 5:**
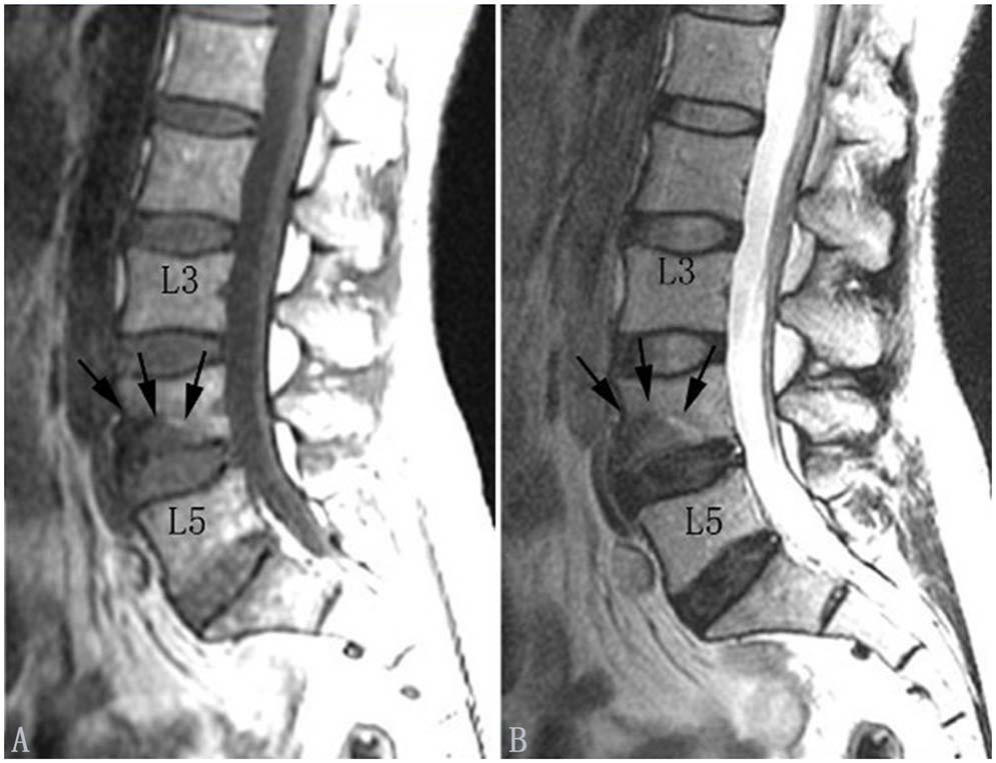
Modic type III changes in the lumbar spine (indicated by arrows). Modic type III changes appear hypointense on T1-weighted (A) and hypointense on T2-weighted images (B).

**Figure 6:**
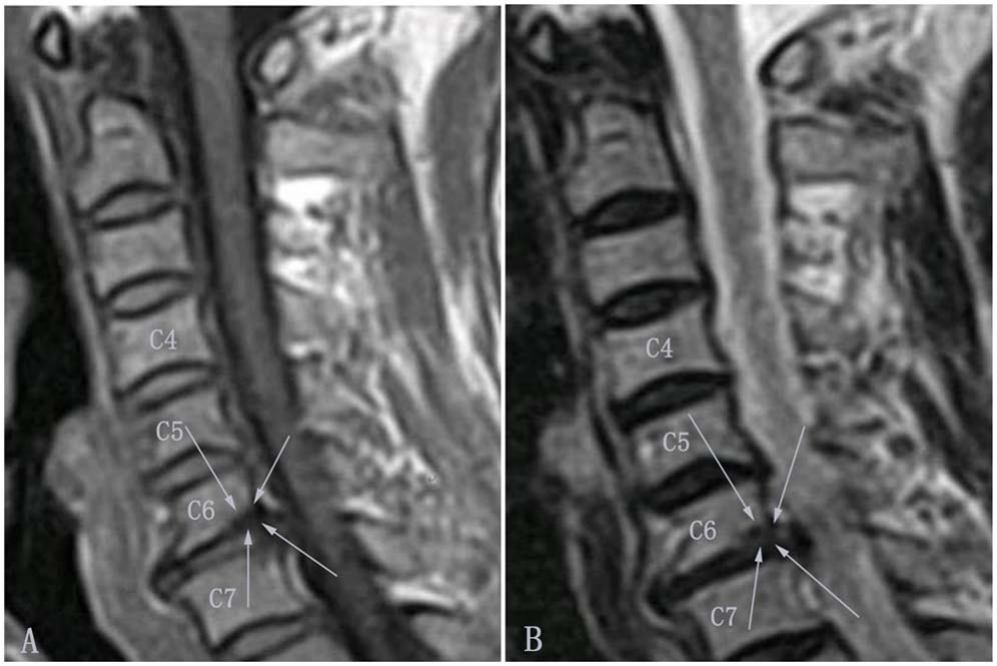
Modic type III changes in the cervical spine (indicated by arrows). Modic type III changes appear hypointense on T1-weighted (A) and hypointense on T2-weighted images (B).

According to the classification criteria of MCs [1]–[3], there are four categories of endplate change (Table 1). Type 0: the endplate signal is normal; Type I: the endplate shows regions that are hypointense on T1WI and hyperintense on T2WI (Fig. 1 and Fig. 2); Type II: the endplate shows regions that are hyperintense on T1WI and isointense or hyperintense on T2WI (Fig. 3 and Fig. 4), but the signal changes are less marked than for Type I; Type III: the endplate shows regions that are hypointense on both T1WI and T2WI (Fig. 5 and Fig. 6). The pathological change associated with Type I MCs is endplate neovascularity or edema. Type II MCs reflect fatty change or necrosis of the endplate, and Type III represent endplate sclerosis.

#### Grade of disc degeneration

Intervertebral disc degeneration was classified into five grades based on the T2-weighted MRI, according to the criteria of Pfirrmann [13].

#### Spinal curvature (lordosis)

**Figure 7:**
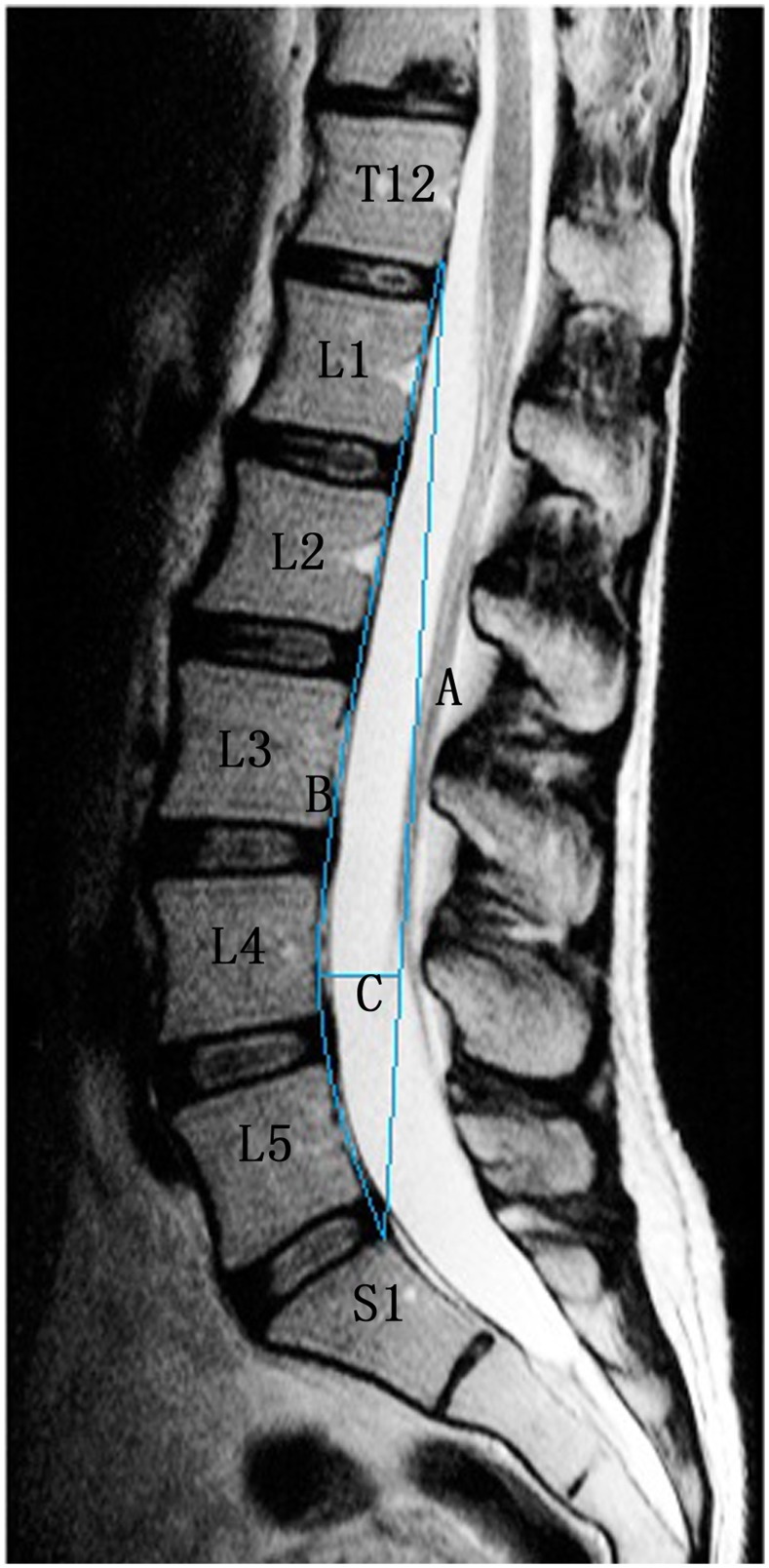
Seze method. A straight line is drawn from the superior posterior aspect of the odontoid process of the T12 vertebra to the posterior inferior aspect of the body of the S1 vertebra named line A. Another line is traced along the posterior aspect of the intervening lumbar vertebral bodies named line B. The third line intersects A perpendicularly at the point of greatest distance between lines A and B. The length of C recorded in millimeters is the depth of the lumbar lordosis.

Lumbar lordosis was assessed using the Seze method (Fig. 7). In the sagittal plane, a curved line (B) is drawn connecting the posterior edges of all vertebral bodies from L1 to S1, and a straight line (A) is drawn from the inferior posterior edge of T12 to the superior posterior edge of S1. A third line (C) is then drawn perpendicular to line B, at the point where the distance between lines A and B is greatest. The length of line C was then used to evaluate the sagittal curvature of the lumbar spine. According to Seze, the majority of cases fall within the range of 18–22 mm. Compared to normal values, there is lessening or even reversal of the lumbar lordosis in individuals with a value of C<18 mm and a larger lumbar lordosis in individuals with C >22 mm [15]. In this study, values of C below 18 mm were classified as kyphotic and values of 18 mm or above were classified as non-kyphotic.

**Figure 8:**
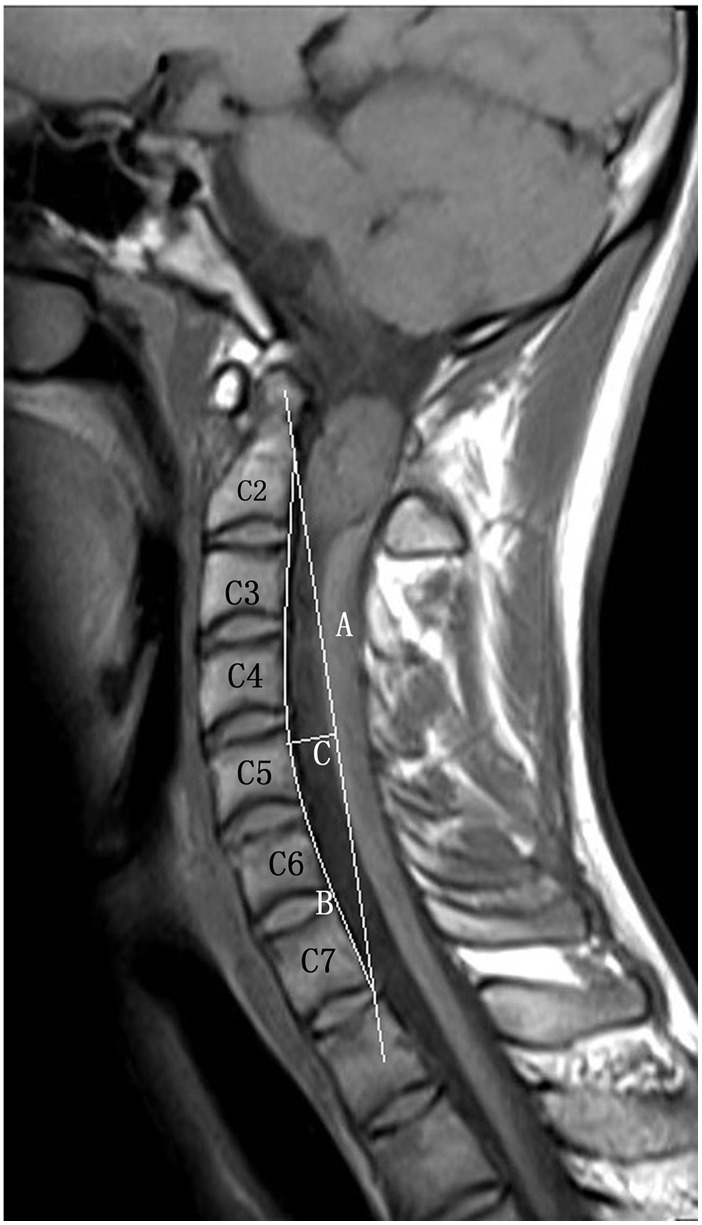
Borden method. A straight line is drawn from the superior posterior aspect of the odontoid process of the C2 vertebra to the posterior inferior aspect of the body of the C7 vertebra named line A. Another line is traced along the posterior aspect of the intervening cervical vertebral bodies named line B. The third line intersects A perpendicularly at the point of greatest distance between lines A and B. The length of C recorded in millimeters is the depth of the cervical lordosis.

Borden’s method was used to measure cervical lordosis (Fig. 8). In the sagittal plane, a curved line (B) is drawn connecting the posterior edges of all vertebral bodies and a straight line (A) is drawn from the superior posterior edge of the odontoid to the inferior posterior edge of the C7 vertebral body. A third line (C) is then drawn perpendicular to line B, at the point where the distance between lines A and B is greatest. The length of line C was then used to evaluate the sagittal curvature of the cervical spine. According to Borden, the majority of cases fall within the range of 7–17 mm. Compared to normal values, there is a lessening or even reversal of the cervical lordosis in individuals with a value of C<7 mm and a larger cervical lordosis in individuals with C >17 mm [16]. In this study, values of C below 7 mm were classified as kyphotic and values of 7 mm or above were classified as non-kyphotic.

### Statistical analysis

The kappa statistic was used to evaluate the inter-observer and intra-observer reliability of the grading of MCs and disc degeneration from MRI. A kappa value of 0.5 was considered to indicate moderate agreement, with a value of 0.8 or higher indicating substantial agreement.

In symptomatic Groups 1 to 3, the prevalence of the three types of MCs at lumbar and cervical levels were compared using the Chi-squared test. Correlations between the prevalence of MCs and disc degeneration, spinal level, and age were assessed using the Spearman rank correlation test, and comparisons of MCs between individuals with kyphotic and non-kyphotic spinal curves were assessed using the Chi-squared test. The Chi-squared test was also used to compare the prevalence of MCs in patients with any back pain (Group 6) or neck pain (Group 7) with the corresponding asymptomatic groups (i.e. Groups 4 and 5, respectively). Statistical software (SPSS 15.0) was used for analysis.

## Results

Kappa values for the intra- and inter-observer analysis of the grading of MCs and disc degeneration were all between 0.74 and 0.89, indicating good to excellent reliability.

The prevalence of the different types of MCs in all patient groups is shown in Table 2. In both lumbar and cervical regions, Type II MCs were most common, occurring in 66–85% of affected endplates in Groups 1 to 5, and Type III were least common, occurring in only 0–6% of affected endplates.

**Table 2:** Prevalence of Modic changes in different patient groups.

Patient Group	Gp 1	Gp 2	Gp 4	Gp 5	Gp 6	Gp 7	Gp 3cervical	Gp 3lumbar
**Number of patients**	1223	1023	304	120	1720	1520	497	497
**Number (%) of** **patients with MCs**	257(21.0)	90 (8.8)	32(10.5)	4 (3.3)	337(19.6)	132(8.7)	42(8.5)	80(16.1)
**Number of endplates**	6115	6138	1520	720	8600	9120	2982	2485
**Number (%) of** **endplates with MCs**	334(5.5)	108(1.8)	48(3.2)	5(0.7)	437(5.1)	152(1.7)	44(1.5)	103(4.1)
**Number (%) of** **Type I**	58(1.0)	35(0.6)	5(0.3)	1(0.1)	77(0.9)	48(0.5)	13(0.4)	19(0.8)
**Number (%) of** **Type II**	273(4.5)	70(1.1)	40(2.6)	4(0.6)	355(4.1)	99(1.1)	29(1.0)	82(3.3)
**Number (%) of** **Type III**	3(0.1)	3(0.1)	3(0.2)	0(0.0)	5(0.1)	5(0.1)	2(0.1)	2(0.1)

### Comparison of the three symptomatic groups (Groups 1 to 3)

The prevalence of MCs in the lumbar spine was slightly higher in the back pain group compared to the back and neck pain group. Similarly, the prevalence of MCs in the cervical spine was slightly higher in the neck pain group compared to the back and neck pain group (Table 2). Based on these findings, symptomatic patients were reclassified into two additional groups: all those with back pain (Group 6) and all those with neck pain (Group 7) in order to simplify the remaining analyses. A comparison of Groups 6 and 7 showed that Type I and II MCs were more common in the lumbar spine of patients with low back pain than in the cervical spine of patients with neck pain (Table 2).

The effects of age, degeneration, and spinal level on the prevalence of cervical and lumbar MCs are shown in Tables 3 and 4 respectively. In both regions, MCs increased with age, grade of disc degeneration, and spinal level. Spearman rank correlations showed that these trends were significant in both regions (Table 5). MCs were also more prevalent in patients with kyphotic (compared to non-kyphotic) spinal curves, in both lumbar (P = 0.00) and cervical (P = 0.00) regions (Table 6).

**Table 3:** Cervical MCs: distribution according to disc degeneration, spinal level and age.

	MCtype	Disc degeneration grade					Spinal level						Age range (yrs)			
		I	II	III	IV	V	C2/3	C3/4	C4/5	C5/6	C6/7	C7/T1	<40	40∼49	50∼59	>60
**Group** **2**	0	102	1334	4320	270	4	1023	1013	1005	971	1000	1018	1933	2268	1267	562
	I	0	0	9	26	0	0	1	6	21	6	1	5	9	14	7
	II	0	4	20	39	7	0	9	12	29	16	4	6	15	25	24
	III	0	0	0	2	1	0	0	0	2	1	0	0	0	2	1
	Total	102	1338	4349	337	12	1023	1023	1023	1023	1023	1023	1944	2292	1308	594
**Group** **3** *	0	654	1165	1055	64	0	497	495	494	480	477	495	1059	1102	582	195
	I	0	1	6	6	0	0	0	1	6	6	0	3	5	4	1
	II	1	4	9	14	1	0	1	2	11	13	2	12	8	7	2
	III	0	0	0	2	0	0	1	0	0	1	0	0	1	1	0
	Total	655	1170	1070	86	1	497	497	497	497	497	497	1074	1116	594	198

*Data from patients in Group 3 with neck pain.

**Table 4:** Lumbar MCs: distribution according to disc degeneration, spinal level and age.

	MCtype	Disc degeneration grade					Spinal level					Age range (yrs)			
		I	II	III	IV	V	L1/2	L2/3	L3/4	L4/5	L5/S1	<40	40 ˜49	50 ˜59	>60
**Group 1**	0	1865	1617	1504	748	47	1212	1203	1192	1126	1048	2015	1867	1149	750
	I	1	9	15	28	5	3	3	8	12	32	14	21	16	7
	II	6	19	75	140	33	7	17	23	85	141	31	91	83	68
	III	0	1	0	2	0	1	0	0	0	2	0	1	2	0
	Total	1872	1646	1594	918	85	1223	1223	1223	1223	1223	2060	1980	1250	725
**Group 3** *	0	412	1393	533	44	0	493	487	482	462	458	863	882	475	162
	I	2	0	9	6	2	0	3	2	6	8	13	10	4	1
	II	0	13	43	22	4	4	6	13	29	30	62	37	15	2
	III	0	0	1	0	1	0	1	0	0	1	1	1	1	0
	Total	414	1406	586	72	7	497	497	497	497	497	895	930	495	165

*Data from patients in Group 3 with back pain.

**Table 5:** Association of Modic changes in various patient groups*.

	Group 1		Group 2	
	Spearman rank correlation	P	Spearman rank correlation	P
**Disc degeneration grade**	0.266	0.000	0.220	0.000
**Spine level**	0.206	0.000	0.087	0.000
**Age range (yrs)**	0.116	0.000	0.217	0.000

*Analyzed by Spearman rank correlation.

**Table 6:** The prevalence of MCs in patients with kyphotic and non-kyphotic spines.

	MC type	Spinal curvature		P	Total
		Kyphotic	Non-kyphotic		
**Group 1: back pain** **only (lumbar levels)**	0	2289	3492	0.00	5781
	I	44	14	0.00	58
	II	185	88	0.00	273
	III	2	1	0.573	3
	MCs total	231	103	0.00	334
	Total	2520	3595		6115
**Group 3: back and neck** **pain (lumbar levels)**	0	794	1588	0.00	2382
	I	14	5	0.00	19
	II	56	26	0.00	82
	III	2	0	0.05	2
	MCs total	72	31	0.00	103
	Total	866	1619		2485
**Group 2: neck pain** **only (cervical levels)**	0	1864	4166	0.00	6030
	I	31	4	0.00	35
	II	54	16	0.00	70
	III	3	0	0.01	3
	MCs total	88	20	0.00	108
	Total	1952	4186		6138
**Group 3: back and neck** **pain (cervical levels)**	0	989	1949	0.00	2938
	I	8	5	0.03	13
	II	22	7	0.00	29
	III	1	1	0.63	2
	MCs total	31	13	0.00	44
	Total	1020	1962		2982

### Modic changes and pain

As shown in Table 2, the prevalence of any MCs was higher in patients with spinal pain (Groups 6 and 7) than in the corresponding asymptomatic patients (Groups 4 and 5 respectively), in both the lumbar spine (P = 0.00) and cervical spine (P = 0.00).

## Discussion

### Summary of results

In this study, we compared the prevalence of MCs in the lumbar and cervical spines of patients with and without spinal pain, and estimated their association with pain, age, disc degeneration, spinal level and spinal curvature. Type II MCs were most common and Type III were least common in all three patient groups. The prevalence of lumbar MCs in people with back pain was little affected by the presence of concurrent neck pain, and the same was true for cervical MCs in people with neck pain with or without concurrent back pain. The prevalence of lumbar MCs in all patents with back pain was greater than that of cervical MCs in those with neck pain. Both lumbar and cervical MCs increased with disc degeneration and decreasing spinal level, and in both regions, kyphotic spinal curvature was associated with a greater prevalence of MCs (Table 6). According to our results, a causal relationship was not proven but there is a significantly higher prevalence of MCs in patients with back and neck pain.

### Strengths and limitations of the study

As far as the authors are aware, this is the first large scale study comparing lumbar and cervical Modic changes in people with and without spinal pain. The MRIs were evaluated by an experienced radiologist and orthopaedic surgeon which led to good levels of reliability in evaluating the presence and types of MC, and the grading of disc degeneration. The main limitation of this cross-sectional study is that it can assess associations only, rather than cause and effect. Cervical MRI scans are not included in routine checkups in China, so our sample size of asymptomatic cervical MRI scans was relatively small.

### Relationship to previous work

Eugen et al [17] [18] reported that MCs in the cervical spine are a dynamic phenomenon [17] that is associated with disc degeneration [18]. In our study also, MCs were positively associated with disc degeneration and age, supporting this previous research.

### Explanation of results

Two aspects are important in the origins of MCs: biomechanics and biochemistry. The spine is a nonlinear viscoelastic structure, and the vertebral endplates comprise its ‘weak link’. Axial (compressive) loading deforms the bone and cartilage endplates, and excessive or repetitive loading may result in irreversible injury. MCs may arise from this mechanism. Compared to superincumbent body weight, the cervical spine is relatively weak in bending, but relatively strong in compression [19]. This could explain why the prevalence of MCs is lower in the cervical spine compared to the lumbar.

Endplates play an important role in disc nutrient transport [20], so endplate damage followed by calcification could reduce this transport and accelerate the process of disc degeneration. Endplate damage also decompresses the adjacent disc [21] and has been shown to cause disc degeneration in animal models [22]. Either mechanism could explain why MCs were associated with increased age and disc degeneration in the present study.

The association between MCs and increased kyphosis can be explained by reduced vertical shock absorption in a flattened spine. This would increase the risk of endplate damage during incidents such as a fall on the buttocks. Wu et al. have shown a *higher* prevalence of MCs in patients with degenerative scoliosis [23], but this frontal-plane spinal curvature is primarily structural and can not be equated with sagittal plane curvature (lordosis) which is more functional than structural.

The results of our study suggest that disc degeneration is associated with MCs, both in the lumbar and cervical spine. However, the exact relationship between MCs and disc degeneration was not clarified and we cannot conclude that disc degeneration results in MCs or *vice versa*. The influence of spinal level on the occurrence of MCs could be due to increased disc degeneration at lower levels, but other explanations are possible. Associations between MCs and age may also be related to disc degeneration [24]–[28].

### Clinical relevance

Although it is one of the most common disorders of middle and old age, low back/neck pain is often difficult to explain in terms of pathological anatomy. Most (almost 80%) is classified as non-specific pain, which can be unsatisfying for both patients and surgeons. Therefore, a causal relationship was not proven but there is a significantly higher prevalence of MCs in patients with back and neck pain.

Normal spine curvature is a manifestation of biomechanical balance, and increased kyphosis can be an early indication of degenerative disease in both the lumbar and cervical spine. Postural education could possibly help to maintain normal lordosis, and reduce the risk of endplate damage, MCs, and pain.

### Suggestions for future work

Our sample size of asymptomatic cervical patients is relatively small and we will expand our data base to investigate more accurately the prevalence of cervical MCs in asymptomatic patients in the future.

## Conclusion

In patients with back or neck pain, MCs of the lumbar endplate mainly occurred at L5/S1, and then at L4/5, while those affecting the cervical endplate mostly occurred at C5/6, then at C4/5 and C6/7. The prevalence of MCs in the lumbar spine was much higher than in the cervical spine, probably because the latter is less vulnerable to compressive overload. Type II MCs were the most common and Type III the least, both in the lumbar and cervical spine. MCs increased with age, spinal pain, increasing disc degeneration, increased kyphosis, and at lower spinal levels, although the causal mechanisms are not clear.
